# *Drosophila melanogaster* as a Versatile Model for Studying Medically Important Insect Vector-Borne Parasites

**DOI:** 10.3389/fcimb.2022.939813

**Published:** 2022-06-02

**Authors:** Firzan Nainu, Emil Salim, Talha Bin Emran, Rohit Sharma

**Affiliations:** ^1^ Department of Pharmacy, Faculty of Pharmacy, Hasanuddin University, Makassar, Indonesia; ^2^ Department of Pharmacology, Faculty of Pharmacy, Universitas Sumatera Utara, Medan, Indonesia; ^3^ Department of Pharmacy, BGC Trust University Bangladesh, Chittagong, Bangladesh; ^4^ Department of Pharmacy, Faculty of Allied Health Sciences, Daffodil International University, Dhaka, Bangladesh; ^5^ Department of Rasashastra and Bhaishajya Kalpana, Faculty of Ayurveda, Institute of Medical Sciences, Banaras Hindu University, Varanasi, India

**Keywords:** fruit fly, model organism, infection, parasite biology, drug discovery, drug repurposing

## Introduction

In the past decades, insect vector-borne parasitic diseases (VBDs), such as malaria, leishmaniasis, and lymphatic filariasis, have caused a devastating health problem, threatening for over 250 million lives worldwide (Wilson et al., 2020). While the causative parasites of VBDs together with their native insect vectors have been largely documented, much remains unknown. Global efforts are still needed to fully elucidate the parasites’ genes that are playing parts in the infectivity and mechanisms responsible in the modulation of host antiparasitic immune responses. In addition, characterization of important VBDs hallmarks and factors responsible in the vector and host susceptibility to parasitic transmission and infection remains underexplored. Once revealed, such information shall provide a vital role in the improvement of preventive actions to control the spread of VBDs and pharmacotherapeutic strategies to limit the disease burden.

Several insects such as mosquitoes, flies (sand flies, black flies, and tsetse flies), and triatomine bugs, have all been implicated in the transmission of parasites to human (Wilson et al., 2020). While much has been done in the identification of insect(s) suitable for the parasites’ life cycle prior to infection in the human, it remains challenging to carry out experimental investigations in the native insect vectors (Schneider, 2000; Zolfaghari Emameh et al., 2015). Complexities in the vector rearing and the lack of genetic toolbox available for carrying out experimental procedures in the native vectors are two of the most encountered obstacles for anyone working in this field (Schneider, 2000; Zolfaghari Emameh et al., 2015). To address this issue, the use of insect model such as fruit fly *Drosophila melanogaster* to study the parasitic biology and other important knowledge related to the transmission and vector-parasite interaction can be seen as one of the alternative endeavors. In this opinion paper, we would like to propose the use of *D. melanogaster* to study the biology, immunology, and pathogenic characteristics of human parasites, and to some extent other vertebrate parasites, to reflect the situation occurred either during parasites’ lifecycle in the vector or during their infectious phase in their definitive vertebrate host. However, it is important to note that the results obtained from the Drosophila works shall be interpreted with caution once the results are translated into either the parasites’ native vectors or their vertebrate host.

## *Drosophila melanogaster* as a Model Organism to Study Parasites at the Viewpoints of Vector and Host

Fruit fly *D. melanogaster* has been proposed as one of the prominent models to uncover many aspects of infectious diseases (Harnish et al., 2021), including to unravel several inquiries related to parasite biology and vector competence (**Table 1**). However, the application of *D. melanogaster* in the field of parasitology remains strictly limited despite its huge potential. With its superior flexibility for genetic modification (Bier, 2005; Ugur et al., 2016) and high genetic similarities to other insects such as mosquitoes, sand flies, and other vector insects (Schneider, 2000; Zolfaghari Emameh et al., 2015), *D. melanogaster* shall allow a genetically tractable yet robust platform to assess the role of parasites’ proteins in the vector. Indeed, this insect has been extensively used in the exploration of cellular and molecular properties of human pathogens, such as bacteria and viruses, in both *in vitro* and *in vivo* conditions (Hughes et al., 2012; Nainu et al., 2020; Harnish et al., 2021). Based on such notion, it would be possible to conduct similar experiments to examine the pathological role of parasites’ proteins. This is particularly important to provide fundamental insights on the conserved pathogenic mechanisms of the parasites as well as antiparasitic immunity activated in the insect vectors. To achieve this, the use of an *in vivo* two-component GAL4/UAS system will provide a prompt yet efficient and economical platform to study the pathogenic function of parasites’ protein in Drosophila. The binary GAL4/UAS system is generally consist of the *Gal4* gene and UAS (Upstream Activation Sequence) site. The *Gal4* gene encodes *Saccharomyces cerevisiae* GAL4, a transcription factor that will bind to the UAS site which subsequently promote the expression of transgene of interest inserted downstream of the UAS site (Brand and Perrimon, 1993). With the help of this binary system, it is highly feasible to carry out experimental studies to investigate the pathological function of parasites’ protein at the cellular and organismal level at every developmental phases. This is particularly important to reveal how the parasites can interrupt host cellular and physiological function in the insect vectors or even, how the vectors can cope with such condition. Furthermore, the application of diverse genetic tools in Drosophila will help to reveal not only parasites’ protein but also insect cellular factors required for parasite replication. This, in the end, shall drive scientific endeavors to identify novel targets for anti-parasitic therapy by targeting the parasites’ protein or cellular factors present in the vector.

**Table 1:** Examples of insect vector-borne parasites studied using *Drosophila* model.

Parasites	Known vectors	Disease in vertebrate	Experimental systems and obtained results	Lessons learned from *Drosophila* model	Refs
*Brugia malayi*	Mosquito (Mansonia)	Lymphatic filariasis in humans	Bioinformatic and molecular analysis revealed that one *B. malayi* gene, *Bm-nhr-11*, was found to be the orthologue of *D. melanogaster E75* molting control gene.	*Bm-nhr-11*, like its *D. melanogaster* orthologue gene, encodes nuclear receptor proteins that are sex-specific in adult *B. malayi*.	(Crossgrove et al., 2008)
*Dirofilaria immitis*	Mosquito (Aedes, Culex, Anopheles, Mansonia)	Lymphatic filariasis in dogs	Bioinformatic and molecular analysis revealed that one *D. immitis* gene, *Di-nhr-6*, was found to be the orthologue of *D. melanogaster E75* molting control gene.	*Di-nhr-6*, like its *D. melanogaster* orthologue gene, encodes nuclear receptor proteins that are sex-specific in adult *D. immitis*.	(Crossgrove et al., 2008)
*Leishmania amazonensis*	Sand fly	Leishmaniasis in human (cutaneous and mucocutaneous)	*In vivo* infection model of *L. amazonensis* was established in *D. melanogaster.* Subsequent *in vivo* forward genetic screen revealed the role of CD36 in the formation of parasitophorous vacuole that is important for *L. amazonensis* infection.	Drosophila can serve as an *in vivo* model system to investigate host-parasite interaction including in the identification of host factors responsible in the parasitic infection.	(Okuda et al., 2016)
*Leishmania donovani*	Sand fly	Leishmaniasis in human (visceral)	*In vitro* RNAi screening and cell culture method were used. Amastigotes of *L. donovani* was engulfed by Drosophila Schneider 2 (S2) cells. Further experiments revealed *lace* (which encodes a subunit of host SPT) as a key factor in the phagocytic uptake of *L. donovani*.	Experimental approach in *D. melanogaster* have revealed new host factor required in the entry and survival of *L. donovani* amastigotes in the host metazoan cells.	(Peltan et al., 2012)
*Plasmodium falciparum*	Mosquito (Anopheles)	Malaria in human	*D. melanogaster* S2 stable cell line system. This *in vitro* system was used to produce soluble full-length PfRH5 protein, a promising potential antigen for the blood-stage human malaria parasite vaccine.	Drosophila S2 cells as a clinically relevant platform for the generation of ‘hard-to-make’ Plasmodium parasite proteins	(Hjerrild et al., 2016)
*Plasmodium falciparum*	Mosquito (Anopheles)	Malaria in human	Testing the inhibitory effect of *D. melanogaster* antimicrobial peptides on *P. falciparum* growth. *D. melanogaster* Metchnikowin-1 (Mtk-1) and Metchnikowin-2 (Mtk-2) inhibited the growth of *P. falciparum* at low concentrations.	*D. melanogaster* antimicrobial peptides are potential antiparasitic agents to target human parasite.	(Tonk et al., 2019)
*Plasmodium gallinaceum* (a close relative of *P. falciparum*)	Mosquito (Anopheles)	Malaria in chicken	*In vivo* fed and injected (*P. gallinaceum*) methods were used. No infection was detected upon the fed of *P. gallinaceum-*infected blood to Drosophila. However, *P. gallinaceum* was detected in fly hemocoel at 9 days post injection.	Drosophila is a potential surrogate (mosquito) to investigate parasite biology and vector competence.	(Schneider and Shahabuddin, 2000)
*Plasmodium gallinaceum* and *Plasmodium berghei*	Mosquito (Anopheles)	*P. gallinaceum* (malaria in chicken) and *P. berghei* (malaria in mice)	Forward genetic screen (*in vivo*) revealed the presence of 18 mutations that decrease the ability of Drosophila to support the growth of *P. gallinaceum.* Two identified genes were homologues with *A. gambiae* and experimentally vital for *P. berghei* propagation in mice.	Unbiased forward genetics in Drosophila can reveal insect (including mosquito) genes responsible in the successful Plasmodium growth.	(Brandt et al., 2008)
*Trypanosoma cruzi*	Triatomine insect (*Rhodnius prolixus*)	Chagas’ disease	Heterologous expression of TcRjl, *T. cruzi* Ras-related GTPase gene, in Drosophila. Transgene TcRjl expression in Drosophila generates extra wing vein phenotypes.	The authors demonstrated the role of TcRjl in the regulation of MAPK-RTK signaling.	(Dos-Santos et al., 2015)

CD36, cluster of differentiation 36; GTPase, guanosine triphosphate hydrolyase; MAPK, Mitogen-activated protein kinase; RNAi, RNA interference; RTK, receptor tyrosine kinase; S2 cells, Schneider 2 cells; SPT, serine palmitoyltransferase.

*Drosophila melanogaster* has several essential qualities of model organism required to address the important yet unrequited facets of VBDs, especially at the early stage of research. It has small size, rapid life cycle, and easily breed and maintained at lower cost (Pandey and Nichols, 2011). Moreover, with its relatively simple genetics, *D. melanogaster* offers a highly tractable solution yet economical for experiments that requires genetic modifications (Jennings, 2011). Even better, an improved list of mutant and transgenic fly lines relevant for human infectious disease models are already available upon request from multiple stock centers around the world (Pandey and Nichols, 2011; Ugur et al., 2016). Using these fly stocks, researchers may conduct an in-depth study on the function of parasites or host genes of interest in the *in vitro* and *in vivo* situations. These experimental features are surely important and advantageous, especially for researchers with limited access to animal infrastructure and funding.

Genetically, Drosophila shares more than 70% similarity with human diseased gene (Pandey and Nichols, 2011). With its close genetic and proteomic similarities to human, Drosophila might also serve as one of the prospective model organisms in the antiparasitic drug discovery. This is particularly important in the existence of ethical constraints to execute high-throughput investigational study on newly discovered drugs using vertebrate animal models. While this approach has been successfully used for the discovery of novel targets for antiviral drug candidates (Martins et al., 2016), such application remains to be seen in the antiparasitic drug discovery. Plausibly, a relatively straightforward assay to be first conducted is the proof-of-principle investigation for antiparasitic effect by using the already available drugs used in the treatment of respective VBDs. For example, one may assess whether primaquine can provide antimalarial protection to the plasmodium-infected Drosophila, similar to its effect to the human host. Once succeeds, pharmacological and toxicological assessments of new drug candidates as well as drug repurposing efforts to treat VBDs can be done using Drosophila model system.

Certainly, a fair justification needs to be made regarding the use of *D. melanogaster* as an insect vector model or even infected host model in the study of human parasites. Several limitations may present hurdles to the experimental applications. One of the biggest constraints is the apparent differences in the anatomical and physiological profiles between Drosophila and humans (Panayidou et al., 2014; Nainu et al., 2020), which might prevent researchers from finding the most precise answers for their research questions. However, this discrepancy also typical for other animal models, even the vertebrate (mammalian) ones. To cautiously limit such drawback, findings obtained from Drosophila studies shall be subjected for confirmation in the mammalian models prior to verification in humans. This has been done, in part, by several researchers in other relevant studies (Pandey and Nichols, 2011; Panayidou et al., 2014; Martins et al., 2016; Nainu et al., 2017), suggesting that biological functions are conserved from *D. melanogaster* to mammals, despite their anatomical differences. Furthermore, Drosophila lacks adaptive immune responses thus limit its application in the vaccination-related studies. However, it is worth noting that many of the Drosophila’s innate immune responses against pathogens, particularly viruses and bacteria, have been confirmed to be exist in humans (Buchon et al., 2014; Xu and Cherry, 2014; Harnish et al., 2021). Such pathogens-mediated immune responses can serve as an appropriate basis for further investigations, whether the same immune pathways also play roles in the antiparasitic immunity. Despite its limitations, Drosophila shall be a promising complementary model system to explore vector competence, parasites biology and their related behaviors inside the vector, and hopefully at the same time might provide a glimpse of evidence for uncharted antiparasitic immunity of the metazoan host. Using this approach, one may present scientific solutions and encourage the achievement of the 3^rd^ goal of sustainable developmental goals (SDGs), improvement of healthy lives and wellbeing for all people, in an affordable and systematic manner. Nevertheless, careful considerations shall be taken when one decides to use fruit fly in their research to accurately reflect the appropriate use of scientific knowledge and potential clinical application of drug candidates.

## Concluding Remarks and Future Directions

At present, *D. melanogaster* has played a prominent role in the comprehensive investigation of pathogenic human viruses and bacteria, including the outcomes of infection on the metazoan host cells and scientific approaches to mitigate the infection. However, the use of *D. melanogaster* in the study of human parasites remain underexplored. With the accessibility of advanced yet cost-effective research toolbox in Drosophila (Pandey and Nichols, 2011; Ugur et al., 2016) and feasibility of research using human parasites (Schneider and Shahabuddin, 2000; Peltan et al., 2012; Okuda et al., 2016), much information will be gained in no time. Indeed, it is in our belief that *D. melanogaster* shall become a versatile model to explore less-studied topics in the field of human/veterinary parasitology. For instance, by examining the expression pattern of anti-parasitic innate immune genes in Drosophila, upon parasitic challenge, one may help to reveal the function of similar genes in the insect vector. Alternatively, Drosophila can help to reveal parasites genes and/or host factors accountable to the susceptibility of host to infection, as shown in the case of malaria, leishmaniasis, and lymphatic filariasis (**Table 1**). Systematic revelation of parasite pathogenic components (proteins and others) and the interactions of such components with host immunity shall lead to a better approach in diseases management and/or parasite/vector controlling. Surely, with proper research inquiries, *D. melanogaster* will offer a tremendous help in the life-threatening battle against VBDs.

## Author Contributions

FN designed the outline of the manuscript. FN and ES drafted the manuscript. All authors carried out the revision of the manuscript and given the approval for submission.

## Conflict of Interest

The authors declare that the research was conducted in the absence of any commercial or financial relationships that could be construed as a potential conflict of interest.

## Publisher’s Note

All claims expressed in this article are solely those of the authors and do not necessarily represent those of their affiliated organizations, or those of the publisher, the editors and the reviewers. Any product that may be evaluated in this article, or claim that may be made by its manufacturer, is not guaranteed or endorsed by the publisher.
